# Potential of Electrospun Poly(3-hydroxybutyrate)/Collagen Blends for Tissue Engineering Applications

**DOI:** 10.1155/2018/6573947

**Published:** 2018-04-19

**Authors:** Luca Salvatore, Vito Emanuele Carofiglio, Paolo Stufano, Valentina Bonfrate, Emanuela Calò, Stefania Scarlino, Paola Nitti, Domenico Centrone, Mariafrancesca Cascione, Stefano Leporatti, Alessandro Sannino, Christian Demitri, Marta Madaghiele

**Affiliations:** ^1^Department of Engineering for Innovation, University of Salento, Campus Ecotekne, Via per Monteroni, 73100 Lecce, Italy; ^2^Distretto Tecnologico High Tech DHITECH Scarl, Campus Ecotekne, Via per Monteroni, 73100 Lecce, Italy; ^3^EggPlant Srl, Via Don Minzoni 27, 70044 Polignano a Mare, Italy; ^4^Department of Biomedical Sciences and Human Oncology, University of Bari “Aldo Moro”, Piazza Giulio Cesare 11, 70124 Bari, Italy; ^5^Institute of Nanotechnology of the National Research Council (CNR NANOTEC), CNR, Campus Ecotekne, Via per Monteroni, 73100 Lecce, Italy

## Abstract

In this work, tunable nonwoven mats based on poly(3-hydroxybutyrate) (PHB) and type I collagen (Coll) were successfully produced by electrospinning. The PHB/Coll weight ratio (fixed at 100/0, 70/30, and 50/50, resp.) was found to control the morphological, thermal, mechanical, and degradation properties of the mats. Increasing collagen amounts led to larger diameters of the fibers (in the approximate range 600–900 nm), while delaying their thermal decomposition (from 245°C to 262°C). Collagen also accelerated the hydrolytic degradation of the mats upon incubation in aqueous medium at 37°C for 23 days (with final weight losses of 1%, 15%, and 23% for 100/0, 70/30, and 50/50 samples, resp.), as a result of increased mat wettability and reduced PHB crystallinity. Interestingly, 70/30 meshes were the ones displaying the lowest stiffness (~116 MPa; *p* < 0.05 versus 100/0 and 50/50 meshes), while 50/50 samples had an elastic modulus comparable to that of 100/0 ones (~250 MPa), likely due to enhanced physical crosslinking of the collagen chains, at least at high protein amounts. All substrates were also found to allow for good viability and proliferation of murine fibroblasts, up to 6 days of culture. Collectively, the results evidenced the potential of as-spun PHB/Coll meshes for tissue engineering applications.

## 1. Introduction

Electrospinning is a processing technique that allows the production of nonwoven mats of submicrometric polymer fibers, starting from various polymer solutions or blends. The dense fibrous structure of electrospun membranes that can be either randomly organized or preferentially aligned along given directions may simulate the native extracellular matrix (ECM) structure of mammalian tissues and is thus very attractive for application in wound healing, tissue engineering, and regenerative medicine. In addition to proper spatial organization, an ideal ECM-mimicking scaffold should also possess bioactive functional groups able to bind cell ligands and potentially guide cellular behavior towards regeneration patterns. This is why the electrospinning of natural polymers, directly derived from mammalian ECM (e.g., collagen, elastin, and hyaluronic acid), has been increasingly explored over the last 15 years [1–6]. Although electrospun fibers from several ECM components can be successfully synthesized, it is well known that important structural changes, such as protein denaturation, may occur upon electrospinning, mainly due to the aggressive solvent(s) used.

The synthesis of electrospun mats or meshes based on type I collagen, the major constituent of the ECM, has been reported in the literature upon collagen dissolution in fluoroalcohols or dilute acetic acid [1, 6–10]. In any case, significant collagen denaturation has been documented [6, 8]. While denaturation impairs the protein resistance to enzymatic degradation and mechanical loading, thus compromising the stability of electrospun membranes in physiological fluids, recent evidence suggests that denaturation may be particularly useful to promote cellular adhesion, as selected binding sites on collagen chains may be masked or unavailable to cells in the triple helical conformation [11, 12]. Moreover, electrospun collagen has been shown to provide more favorable surfaces for cell adhesion compared to electrospun gelatin, which highlights that electrospun collagen cannot be considered identical to gelatin, likely due to the fact that the polypeptide chains of collagen are not as extensively degraded as those of gelatin [13]. In the overall, collagen denaturation induced by the electrospinning process may positively affect the scaffold bioactivity, provided that the mechanical properties and degradation rate are properly controlled.

Different approaches have been proposed so far to bypass or counteract the effects of electrospinning-induced collagen denaturation, which include the coating of nonwoven mats with collagen right after electrospinning [14, 15], the use of concurrent [4] or terminal crosslinking treatments [1, 2, 5, 6], and/or the electrospinning of collagen in conjunction with other biocompatible and biodegradable polymers, of either synthetic (e.g., polycaprolactone [9], polylactic acid [13], and polyurethane [16]) or natural origin (e.g., chitosan [17], silk fibroin [18], and polyhydroxyalkanoates [7, 10, 19]). With specific regard to crosslinking treatments, it is worth recalling that, as shown by recent investigations [11, 12], the crosslinking reaction(s) may reduce the number of cell-binding domains available to cells, thus adversely decreasing the potential bioactivity of collagen.

Among naturally derived polymers, poly(3-hydroxybutyrate) (PHB), a polyester produced by soil bacteria, algae, and modified plants in stress condition [20], has recently emerged as an appealing, low-cost candidate for the synthesis of various biomedical devices, including controlled release systems, surgical sutures, wound dressings, orthopedic devices, scaffolds for bone tissue engineering, and skin substitutes [21–24]. Several studies indeed demonstrate that PHB has an excellent biocompatibility, providing support to cell attachment and proliferation, especially when used in the form of electrospun, ultrafine fibers with a high surface-to-volume ratio [25, 26]. However, PHB presents various chemophysical issues, such as brittleness, hydrophobicity, lack of specific cell-binding sites, and slow degradation rate. Such limitations are usually dealt with by combining PHB with its more ductile copolymer, poly(3-hydroxybutyrate-co-3-hydroxyvalerate) (PHBV) [19, 25], or with other biocompatible polymers [15, 27].

In this work, we explored the use of several PHB and type I collagen blends (PHB/Coll) for the synthesis of electrospun fibrous meshes with potential for tissue engineering applications. It was hypothesized that the direct combination of PHB and Coll could provide novel substrates with controllable properties for tissue engineering, being each blend component potentially able to mitigate the drawbacks of the other. In particular, we assumed that collagen could provide the electrospun mats with the necessary biomimetic cues and improved wettability, while PHB could control their stiffness and degradation rate, without the need for additional collagen-crosslinking treatments. To test this hypothesis, we fabricated electrospun mats from three different PHB/Coll blends (having weight ratios of 100/0, 70/30, and 50/50, resp.) and characterized their morphological, chemical, thermal, mechanical, and degradation properties. Preliminary cell studies with murine fibroblasts were also performed to evaluate the cytocompatibility of the proposed PHB/Coll substrates.

## 2. Experimental

### 2.1. Materials

The materials used for the synthesis of electrospun meshes were poly(3-hydroxybutyrate) (PHB), provided by Sigma Aldrich (Milan, Italy), and type I collagen (Coll) derived from bovine dermis, supplied by Collagen Solutions Inc. (San Jose, CA, USA). All other chemicals used in this study were purchased from Sigma Aldrich (Milan, Italy) unless otherwise stated and used as received.

### 2.2. Preparation of Nonwoven Mats from PHB/Coll Blends

PHB and type I collagen blends in 1,1,1,3,3,3-hexafluoro-2-isopropanol (HFIP, Thermo Fisher Scientific) were prepared by homogenizing at 15,000 rpm for 10 min at room temperature (VELP Ov5 homogenizer). The so-prepared cloudy blends had a final polymer concentration of 2% (*w/v*), with weight ratios between PHB and collagen (PHB/Coll) equal to 100/0, 70/30, and 50/50, respectively. The blends were then kept under magnetic stirring overnight at room temperature, before further processing. Electrospinning was performed at room temperature and relative humidity RH = 40–50% in a horizontal spinning configuration, by using an equipment provided by Linari Engineering (Italy). A glass syringe containing the PHB/Coll blend (15 mL) was connected to a stainless steel blunt-ended needle (inner diameter 0.8 mm). A rotating collector (with a diameter of 60 mm and a rotational speed of 300 rpm) was covered with aluminum foil and positioned at a given distance from the tip of the needle (in the range 12–24 cm). Deposition of electrospun fibrous mats was obtained with a constant feed rate of 0.03 mL/min, under an applied voltage-processing range from 12 to 16 kV.  Table 1 summarizes the specific electrospinning conditions used for the different PHB/Coll blends.

### 2.3. Morphological Analyses

The surface morphology of the samples was firstly analyzed by means of scanning electron microscopy (SEM). Briefly, small discs (8 mm diameter) were punched out from each electrospun mat, placed onto the sample holder, and observed without any further manipulation by using a Zeiss EVO microscope (variable pressure mode, accelerating voltage of 20 kV). The obtained micrographs were then processed and analyzed by means of the line selection tool of ImageJ software (National Institutes of Health, USA), to estimate the average fiber diameter and size distribution over 100 different fibers. ImageJ was also used to determine the value of porosity (*P*) at three different layers or depths within the thickness of the electrospun membranes (i.e., surface, middle, and total thickness with porosity *P*_1_, *P*_2_, and *P*_3_, resp.), upon appropriate thresholding of the SEM micrographs, according to the method described by Ghasemi-Mobarakeh et al. [28]:

(1) $$\begin{array}{c} P_{i}=\left(1-\frac{n_{i}}{N_{i}}\right)\times 100. \end{array}$$

In the above equation, *n*_*i*_ is the number of white pixels (i.e., pixels associated to fibers) and *N*_*i*_ is the number of total pixels in the binary image associated to the given layer *i*. A total of 3-4 SEM micrographs were analyzed for each sample type.

Further information on the surface morphology of as-spun meshes was acquired by means of atomic force microscopy (AFM). All acquisitions were conducted in air at room temperature (RT) using a Nanoscope V Picoforce Multimode Scanning Probe workstation (Digital Instruments, Santa Barbara, CA) operating in tapping mode. Scans were performed using R FESPA tip (Bruker) at high resolution (512^∗^512) on different scales (50 *μ*m^∗^50 *μ*m, 20 *μ*m^∗^20 *μ*m) and a low scan rate (0.3 Hz). AFM images were finally analyzed with ImageJ software to evaluate the fiber size distribution over 100 different fibers, as described above for the SEM micrographs.

### 2.4. Chemical Composition and Wettability

The chemical composition of PHB/Coll membranes was assessed by means of Fourier transform infrared (FTIR) spectroscopy (FTIR-6300, Jasco), in order to verify the presence of both PHB and collagen in the composite samples. All spectra were recorded on KBr pellets in the range of 4000 to 400 cm^−1^, with a resolution of 4 cm^−1^ and averaged over 64 scans.

Moreover, the membrane wettability was evaluated by means of contact angle measurements performed by a sessile drop method (FTA1000 equipment). The static water contact angle (WCA) was measured by placing a drop of approximately 40 *μ*L on the sample surface. At least 4 readings were performed for each specimen.

### 2.5. Thermal Analyses

Thermal stability of starting materials (PHB and collagen) and as-spun mats (in dry state) was assessed by thermogravimetric analysis (TGA, Mettler Toledo). Samples (5–15 mg) were placed in alumina crucibles, maintained at 25°C for 5 minutes, and then heated up to 800°C at a constant rate of 10°C/min. Measurements were carried out under 50 mL/min of nitrogen flow, using an empty crucible as a reference, and run in triplicate. The first derivative of the TGA signal (DTG) was also calculated to assess the mass loss rate.

The melting transition of the PHB component in the electrospun mats was then analyzed by means of differential scanning calorimetry (DSC, TA Instruments). Dry samples (5–15 mg, *n* = 3) were placed in aluminum pans and heated from 4°C to 200°C at 10°C/min under a 50 mL/min nitrogen purge, using an empty pan as a reference. The crystallinity degree (*χ*) was calculated according to the following:

(2) $$\begin{array}{c} \chi =\frac{\Delta H_{m}}{\left(\Delta H_{m100\%}*W_{PHB}\right)}, \end{array}$$

where Δ*H*_m_ is the apparent melting enthalpy of PHB, Δ*H*_m100%_ is the extrapolated melting enthalpy of 100% crystalline polymer (146 J/g [21]), and *W*_PHB_ is the PHB weight fraction in the blend [29]. Raw PHB powder was also analyzed and compared to pure PHB mats to detect any effect of the electrospinning process on polymer crystallization and melting.

### 2.6. Mechanical Tests

Tensile properties of dry PHB/Coll membranes were assessed by means of a universal testing machine (Zwick Roell, Germany) equipped with a 100 N load cell, under displacement control (displacement rate = 0.1 mm/s). During the test, the load and displacement were monitored and recorded. Five rectangular samples from each group (10 × 60 mm) were tested to obtain the average Young's modulus (*E*), calculated by the slope of the linear elastic region of the stress-strain curve at low strain values (in the range 0–2%). Elongation at break (*ε*_b_) and tensile strength (*σ*_max_) were also evaluated.

### 2.7. In Vitro Hydrolytic Degradation

The hydrolytic degradation of PHB/Coll meshes was monitored by means of gravimetric measurements. Briefly, square samples (about 5 mg each) were placed into 15 mL of phosphate-buffered saline (PBS) and incubated in a thermostatic bath at 37°C to simulate physiological conditions. Every two days, the PBS was replaced with fresh one. At selected time points (1, 4, 8, 14, and 23 days), samples were recovered, washed with distilled water, dehydrated in a vacuum oven (30°C) for 5 hours, and weighed again. The degradation extent over time was then calculated according to the following:

(3) $$\begin{array}{c} D_{hydrolytic}=\left(1-\frac{W_{t}}{W_{0}}\right)\times 100, \end{array}$$

where *W*_0_ is the initial sample weight and *W*_*t*_ is the weight at time *t*. At each time point, measurements were performed in triplicate.

### 2.8. Cell Culture Experiments

#### 2.8.1. Cell Proliferation (MTT Assay)

Cell proliferation on PHB/Coll samples was evaluated *in vitro* by the thiazolyl blue tetrazolium bromide (MTT) assay. The assay is based on the ability of viable cells to reduce the yellow, water-soluble tetrazolium salt to a purple, water-insoluble formazan product. The formazan crystals are produced by dehydrogenases in active mitochondria of viable cells. The MTT assay thus provides an indication of metabolic activity, which is interpreted as a measure of cell viability. Briefly, the fibrous mats were cut into discs (8 mm diameter), sterilized by means of dry heat (121°C for 3 hours under vacuum), and hydrated in PBS solution. Subsequently, samples were transferred into a 24-well culture plate. Mouse fibroblast cells (NIH 3T3) were seeded onto each sample at a density of 2.5 × 10^4^ cells/well. Cells were allowed to adhere to the samples for 1 hour, and then 1 mL of culture medium (Dulbecco's Modified Eagle's Medium, DMEM) supplemented with 10% (*v/v*) fetal bovine serum (FBS), 1% L-glutamine, and 1% penicillin/streptomycin was added. Cells seeded onto cover slides appropriately treated for cell culture were used as a control. The plate was incubated in humidified atmosphere with 5% CO_2_ at 37°C. At selected time points (1, 3, and 6 days), 50 *μ*L of MTT solution (5 mg/mL) was added to the medium and samples were incubated for further 4 h. At the end of incubation, the medium was removed and purple formazan crystals were solubilized with 1 mL of acid isopropyl alcohol (0.04 M HCl in isopropyl alcohol) at room temperature. The optical density of the formazan solution was read on a spectrophotometer at a wavelength of 550 nm. The obtained absorbance values were compared to those of control cells at day 1 and expressed as percentage of cell viability/proliferation. All tests were performed in triplicate and repeated twice.

#### 2.8.2. Cell Adhesion and Viability (Live/Dead Staining)

Additional information on initial cell attachment and viability on the fibrous PHB/Coll meshes was obtained by a live/dead staining performed with fluorescein diacetate (FDA) and propidium iodide (PI), which stain viable and nonviable cells, respectively. The FDA + PI assay provides useful information regarding both the physiologic state and the membrane integrity of cells, as FDA is cleaved by nonspecific esterases in the cytoplasm of viable cells to liberate a green fluorescent molecule, while PI, a red fluorescent intercalating dye, only passes through the membranes of dead or dying cells [30]. Briefly, sterile membrane discs were transferred into a 24-well culture plate and 3T3 cells were seeded onto each disc, at a density of 5 × 10^4^ cells/well. After 24 hours, the medium was removed and the samples were washed twice with 1 mL of PBS for 5 minutes, under gentle agitation. Successively, 1 mL of PBS and 10 *μ*L of freshly prepared FDA solution (0.5 mg/mL in acetone) were added onto each sample. After 15 minutes, 10 *μ*L PI solution (2 mg/mL in phosphate-buffered saline without CaCl_2_ and MgCl_2_) was also added. The specimens were then incubated for 45 minutes at 22°C, before being washed twice with 1 mL of PBS for 15 minutes. The evaluation of cell viability was finally conducted by means of confocal microscopy, by visualizing viable (green) and nonviable (red) cell populations.

### 2.9. Statistical Analysis

All results were expressed as mean ± standard deviation. The effect of different PHB/Coll ratios on the properties of the fibrous membranes was assessed by means of one-way ANOVA, while Fisher's PLSD tests were used to compare individual sets of data. A *p* value < 0.05 was considered significant.

## 3. Results and Discussion

### 3.1. Morphology of Electrospun Meshes

**Figure 1:**
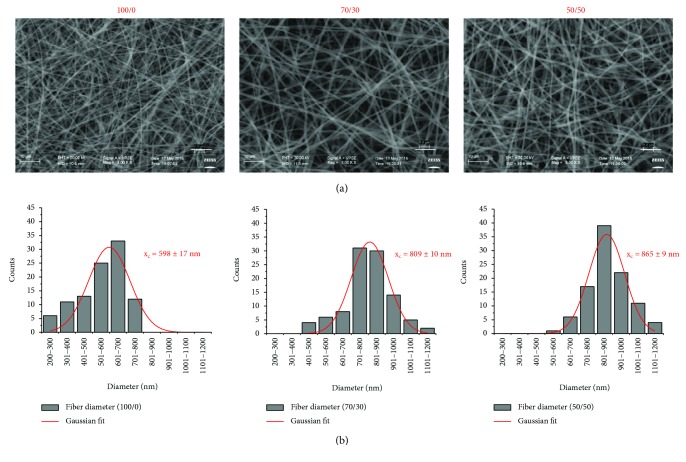
Representative SEM micrographs (3000x, scale bar 10 *μ*m) of electrospun meshes with different PHB/Coll ratios (a) and corresponding fiber size distributions (average values and standard deviation over 100 fibers, with Gaussian fit) (b).

The electrospinning of several PHB/Coll blends (2% *w/v* in HFIP), differing for the PHB/Coll weight ratio (100/0, 70/30, and 50/50), was successfully performed by empirically adjusting the applied voltage and/or the collection distance (i.e., the distance between the tip of the needle and the surface of the rotating collector) as a function of the given PHB/Coll blend being processed. By using the parameters listed in Table 1, nonwoven fibrous mats or membranes, with an average thickness of approximately 35–50 *μ*m and a macroscopically smooth morphology, could be obtained. Detailed examination by SEM at different magnifications highlighted a homogeneous web-like structure devoid of beads, with randomly oriented submicrometric fibers. Further analysis of the micrographs through the use of ImageJ software allowed estimating the average fiber size and fiber size distribution for each type of membrane (Figure 1). Of note was that the average fiber diameter was found to increase as the collagen fraction in the initial polymer blend increased: mean fiber sizes of 598 ± 17 nm, 809 ± 10 nm, and 865 ± 9 nm, estimated by Gaussian fitting of the fiber size distribution, were indeed detected for PHB/Coll ratios of 100/0, 70/30, and 50/50, respectively. Accordingly, a fiber size distribution within the range 200–800 nm was obtained for pure PHB membranes, while larger distributions, from 400 to 1200 nm, were yielded for composite ones. These findings were likely ascribed to the higher viscosity of the PHB/Coll blend resulting from increased collagen amounts and appeared in agreement with previous data on the synthesis of electrospun PDLLA/collagen fibers [13]. However, it is worth mentioning that an opposite effect of the collagen content on the fiber size has also been documented in the literature for PHBV/collagen blends [10]. The reason for such variable findings probably lies in the different viscosities yielded by the two polymers in the blend, at fixed concentration. It is well known that viscosity is a key parameter affecting the ejection and the jet/fiber thinning under the electrostatic field, with smaller fibers yielded by less viscous polymer solutions [1, 19, 31, 32]. As such, the solvent, the molecular weight of the polymer(s), the total polymer concentration, and/or the blend composition are all variables that influence the synthesis of electrospun fibers of given size. In the case of collagen, the specific animal tissue of origin and the extraction protocol will further affect the final viscosity of the polymer solution [1], and this clearly contributes to make results from independent investigations hardly comparable. Nonetheless, the possibility to control the fiber diameter by adjusting the blend composition (i.e., the PHB/Coll ratio) represents a simple way to modulate the surface area of the electrospun meshes, thus tailoring them to specific biomedical applications, for example, tissue engineering and wound healing, for which the promotion of cell-material interactions is highly desirable.

Further analysis of the SEM micrographs was also performed to estimate the average porosity of the as-spun membranes at different depths along their thickness, as previously reported [28]. While other techniques (e.g., gravimetric measurements and mercury porosimetry) provide an estimation of the total porosity of given three-dimensional scaffolds, the method described by Ghasemi-Mobarakeh et al. [28] is specifically directed to the study of multilayered porous membranes and offers the advantage of discerning the surface porosity from the bulk one. In particular, decreasing values of porosity are expected when moving towards deeper layers, due to the increasing number of overlapping fibers. As summarized in Table 2, all of the samples showed values of surface (*P*_1_), middle (*P*_2_), and total (*P*_3_) porosities of about 84%, 50%, and 18%, respectively. However, 70/30 samples displayed significantly higher values of *P*_2_ and *P*_3_ when compared to 100/0 (*p* = 0.03 for *P*_2_ and *p* = 0.003 for *P*_3_) and 50/50 (*p* = 0.038 for *P*_2_ and *p* = 0.027 for *P*_3_). As regard to the surface porosity *P*_1_, the only significant difference was the one detected between samples 50/50 and 100/0, with the former being less porous than the latter (*p* = 0.03).

These findings suggested that the PHB/Coll weight ratio in the polymer blend does affect not only the average fiber size but also the overall porosity of the as-spun meshes. In particular, the middle (*P*_2_) and total porosities (*P*_3_) are greatly affected by the PHB/Coll ratio, being maximized for an intermediate ratio of 70/30. This finding is consistent with the higher thickness of the 70/30 samples compared to the others (Table 2). On the contrary, the surface porosity (*P*_1_) is slightly influenced by the PHB/Coll ratio and appears to be related to the average fiber size that tends to decrease as the fiber diameter is increased. The high values of *P*_1_ here reported are in accordance with previous studies on different types of electrospun mats [13, 28] and suggest that a highly porous surface is potentially available to accommodate seeded or incoming cells. However, it is worth noting that fiber swelling in water solutions, especially in the presence of increasing fractions of hydrophilic collagen, is likely to occur, as reported for other types of electrospun membranes [13] and this may significantly reduce the overall porosity available for cell colonization.

**Figure 2:**
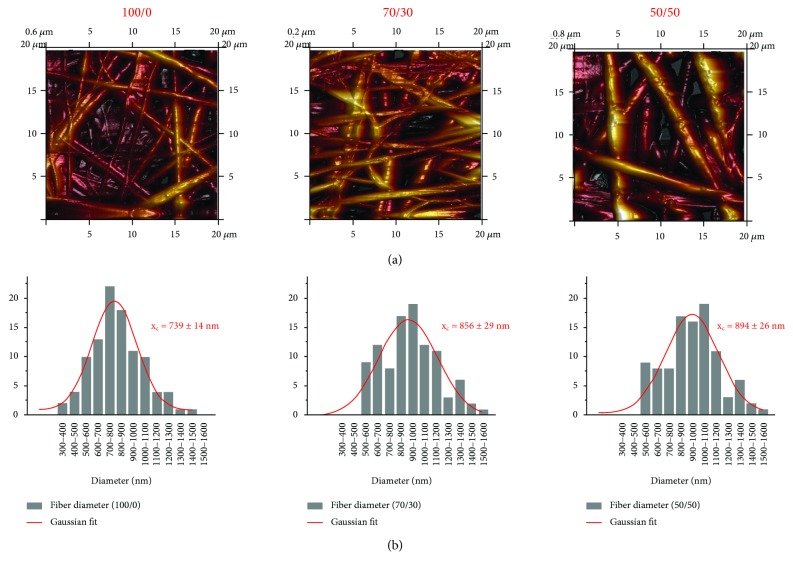
Representative AFM images of electrospun meshes with different PHB/Coll ratios (a) and corresponding fiber size distributions (average values and standard deviation over 100 fibers, with Gaussian fit) (b).

AFM analysis on the as-spun membranes further showed the random organizations of polymeric fiber networks (Figure 2). In addition, the analysis on 100 different fibers confirmed the trend obtained in electron microscopy, demonstrating the increase of the average value of fiber diameter with the collagen concentration. By fitting the results of the analysis with Gaussian distribution, the fiber diameter was found to change from 739 ± 14 nm to 856 ± 29 nm and to 894 ± 26 nm for PHB/Coll ratios of 100/0, 70/30, and 50/50, respectively.

### 3.2. Chemical Composition and Wettability

**Figure 3:**
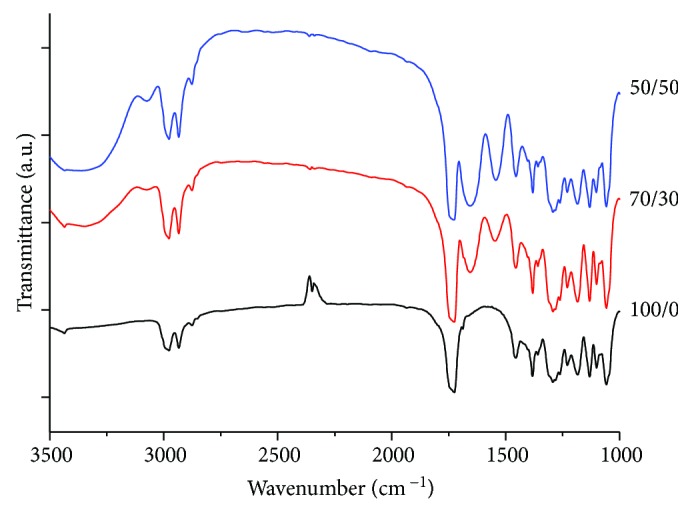
FTIR spectra of the PHB/Coll meshes, plotted in the range 3500–1000 cm^−1^. Amide bands ascribable to collagen are clearly visible in 70/30 and 50/50 samples, at 3321 cm^−1^, 3079 cm^−1^, 1640 cm^−1^, and 1545 cm^−1^.

The chemical composition of fibrous meshes was assessed through FTIR (Figure 3). As expected, the spectrum of pure PHB fibers (i.e., PHB/Coll 100/0) showed a strong and characteristic absorption band at 1726 cm^−1^, ascribable to the carbonyl stretching (C=O), and a slight absorption at 3440 cm^−1^, representative of hydroxyl groups [10, 33]. Further bands in the ranges 1292–1456 cm^−1^ and 1043–1181 cm^−1^, associated to CH and CO vibrational modes, respectively, were also detected, in accordance with the literature [10, 21, 33, 34]. On the other hand, the spectra of composite PHB/Coll fibers (with weight ratios of 70/30 and 50/50) distinctly showed additional amide bands related to the protein, such as amide A (3321 cm^−1^, due to NH stretching), amide B (3079 cm^−1^, due to CH stretching), amide I (1640 cm^−1^, due to CO stretching), and amide II (1545 cm^−1^, due to NH bending and CN stretching). Obviously, the absorbance of these bands was found to increase with the collagen content, due to the increased amount of available amino groups [7, 10]. This demonstrated the successful incorporation of collagen within the composite fibers.

The static water contact angle (WCA) was also evaluated to investigate the effect of collagen on the mat wettability. Surface wettability is indeed critical for biomedical applications, as cells better adhere, spread, and grow on moderately hydrophilic substrates rather than on very hydrophobic or very hydrophilic ones [35]. As expected, while PHB membranes were highly hydrophobic, with a WCA of about 119°, composite PHB/Coll ones showed a much higher wettability, with average WCA values in the range 39°–64° (Table 2). Of note was that the 70/30 samples appeared to be less hydrophobic than the 50/50 ones, in spite of their higher PHB content, although a very high variability of the static WCA was detected. In general, the hydrophobic/hydrophilic character of a substrate can be attributed to the presence of specific chemical groups on the surface, as well as to the surface roughness. Although the electrospinning process may induce a certain rearrangement of chemical moieties on the fiber surface [35], the apparently increased wettability of the 70/30 membranes may be ascribed to their surface porosity (*P*_1_) and/or roughness. A previous study on electrospun PLGA mats showed that the substrate porosity might enhance its hydrophilicity [35]. Furthermore, previous independent investigations on fibrous mats versus smooth films, based on PHB and PHBV, suggested that an increased surface roughness tends to decrease the substrate wettability, by introducing multiple contact points with the water droplet that alter the water-polymer interface [25, 34].

### 3.3. Thermal Properties

**Figure 4:**
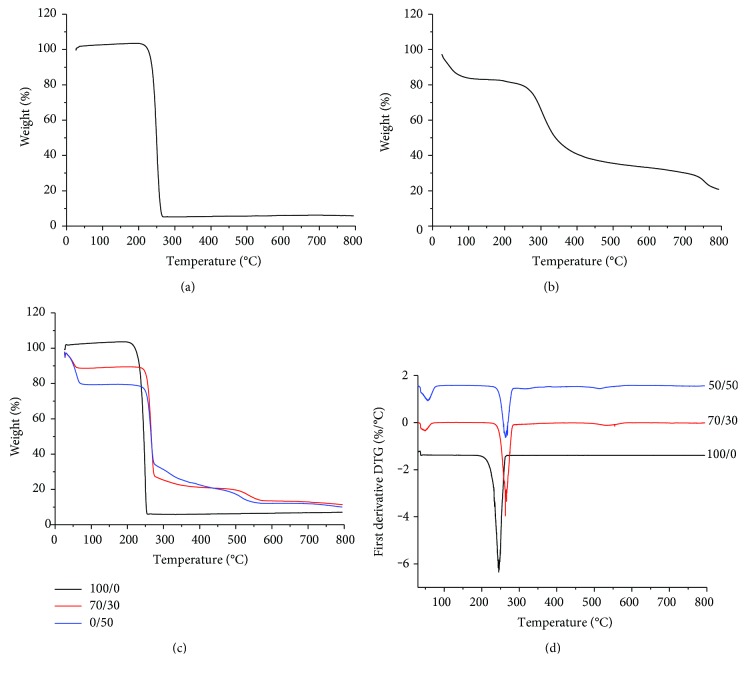
TGA curves of PHB powder (a) and type I collagen flakes (b) used for the synthesis of the fibrous meshes; TGA (c) and DTG curves (d) of the blended fibrous samples, as a function of the PHB/Coll ratio.

Thermogravimetric analysis (TGA) was performed on both blend precursors (i.e., raw PHB powder and collagen flakes) and electrospun PHB/Coll meshes, to verify the effect of each component on the thermal stability of the blends. Experimental results showed that PHB powder and PHB fibrous mats (Figures 4(a) and 4(c), resp.) displayed a similar behavior, with a fast, single-step weight loss of approximately 95%, which is representative of the thermal degradation of the polymer [34, 36]. The thermal stability of the powder was found to be only slightly higher than that of fibers, with a maximum mass loss rate detected at 250°C and 245°C, respectively (Figure 4(d)). This was likely ascribed to the increased surface area of the fibrous mats, which contributed to accelerate the PHB degradation [34]. With regard to the polymer blends, TGA results showed that PHB/Coll mats had an increased thermal stability with respect to PHB ones (Figures 4(c) and 4(d)). Composite fibrous mats indeed showed a gradual weight loss, occurring in three different steps. A slight mass loss (about 12–20%) in the temperature range 25–100°C was recorded as a result of the evaporation of unbound water (i.e., humidity) absorbed by the collagen [37]. This weight loss appeared proportional to the amount of collagen in the composite samples (12% and 20% for PHB/Coll mats with weight ratios of 70/30 and 50/50, resp.) and was also in rough accordance with the average value of humidity recorded for the raw collagen flakes (Figure 4(b)). A sharp and fast mass loss, corresponding to 63% and 46% for samples with PHB/Coll equal to 70/30 and 50/50, respectively, was then detected at about 262°C. Such a mass loss, proportional to the PHB content, was mostly related to PHB degradation, although concurrent and partial collagen decomposition was also likely to occur. The three-step thermogram of raw collagen flakes indeed suggested that thermal degradation of collagen, due to partial decomposition of covalent bonds, starts at temperatures higher than 200°C. Composite PHB/Coll fibrous mats then showed a terminal mass loss at around 500°C, related to complete degradation.

**Figure 5:**
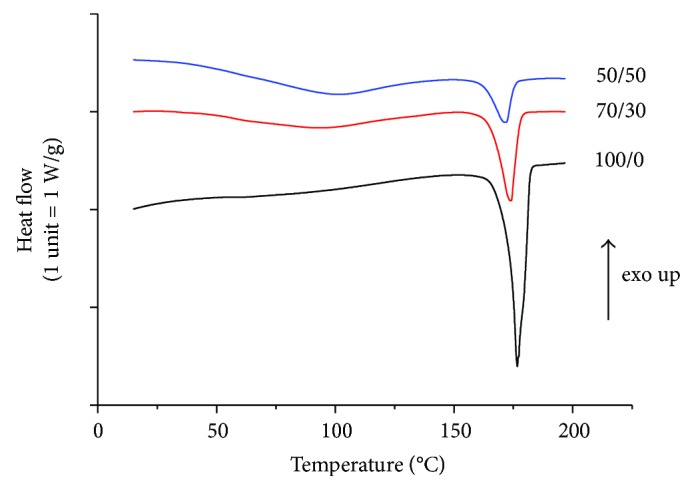
DSC curves of fibrous samples: effect of the PHB/Coll ratio on PHB melting.

Differential scanning calorimetry (DSC) was also performed to further evaluate the interaction between the two blend components, by analyzing the effect of collagen on PHB melting (Figure 5). As expected, the sample composition was found to significantly affect both the melting temperature (*T*_m_) and the melting enthalpy (Δ*H*_m_) (*p* = 0.0001 and *p* = 0.0004, resp.). Electrospun PHB fibers had *T*_m_ of about 176°C and Δ*H*_m_ of 94 J/g, in rough agreement with the literature (Table 3) [10, 19, 21]. Interestingly, such values were higher than the ones recorded for the raw PHB powder, although the difference was significant only for *T*_m_ (*p* = 0.04 and *p* = 0.18 for *T*_m_ and Δ*H*_m_, resp.). An increase of the melting temperature and/or enthalpy upon electrospinning, together with an increase of the glass transition temperature (*T*_g_), has been reported in the literature for several polymers, including PHB, as a result of restricted chain mobility and/or increased polymer orientation in the fiber direction [21, 34]. However, in this study, the electrospinning process itself seemed to have only a very slight effect on the melting transition of PHB.

Of note, composite PHB/Coll fibrous membranes showed significantly decreasing *T*_m_ values as the collagen content was increased, with *T*_m_ equal to 173.5°C and 171.8°C for PHB/Coll ratios of 70/30 and 50/50, respectively (*p* = 0.009). This melting point decrease may be associated to the formation of smaller PHB crystals in the polymer blend (i.e., the reduction of lamellar thickness) and/or to physical interactions between the two polymers in the amorphous regions [21].

The melting enthalpy (Δ*H*_m_) was also found to significantly decrease with the collagen content (Table 3), similarly to what has been reported in the literature for other PHB-based blends [10, 19, 21]. This finding was clearly correlated with a lower degree of crystallinity attained by PHB in the presence of collagen. In the overall, the DSC data showed that the addition of collagen to PHB upon electrospinning induces the formation of less crystalline mats, which are thus expected to be less fragile and more rapidly degradable than PHB ones [14].

### 3.4. Mechanical Properties

Tensile tests were performed to evaluate the mechanical properties of as-spun PHB/Coll meshes in the dry state and particularly to ascertain whether the addition of collagen could mitigate the typical rigidity and brittleness of PHB (ascribable to its highly crystalline nature) that often limit its practical applications [27]. Independently of the PHB/Coll ratio, we observed that all samples displayed a similar deformation mechanism upon loading, with a certain degree of lateral contraction, as expected, but no evident necking phenomenon. It has been shown that lateral contraction of nonwoven mats is ascribable to the buckling of fibers that are initially perpendicular or laterally oriented with respect to the loading direction, whereas fibers oriented along the loading axis undergo further alignment [38]. The mechanical properties of fibrous membranes thus depend on both the mechanical properties of the single fibers making up the membrane and the structure of the membrane itself, for example, how tight the fibers are packed and interconnected, and whether the fibers show any preferential orientation [38–40].

**Figure 6:**
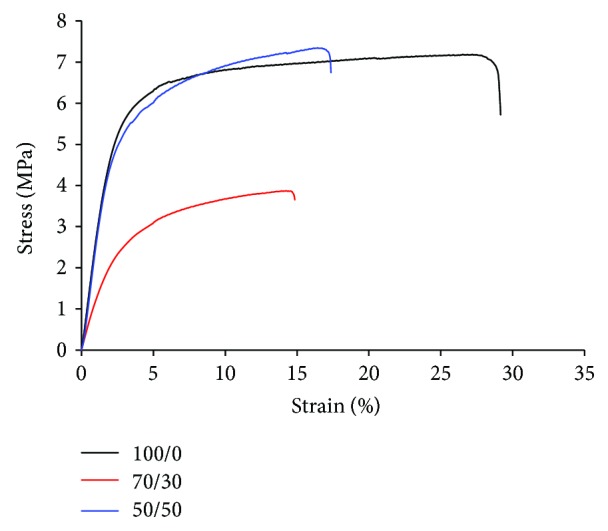
Typical tensile stress-strain curves of the PHB/Coll meshes.

The stress-strain curves obtained for the different types of PHB/Coll meshes are reported in Figure 6. For semicrystalline polymers such as PHB, the tensile response of single fibers at low strains can be ascribed to the elongation of the polymer chains in the interlamellar, amorphous domains, as well as to the intralamellar separation of crystalline block segments. In this regard, crystallites act as fillers or crosslinks that stiffen and strengthen the polymer chains, so that higher elastic moduli and higher strengths are expected for increasing crystallinity degrees. At larger strains, both amorphous polymer chains and crystalline block segments can slide and align along the stress direction, until rupture occurs [41]. This deformation mechanism appears in accordance with the measured profile of the stress-strain curves (Figure 6), where the very gradual stress increase at large strain is attributable to chain sliding and orientation.

As summarized in Table 4, all samples displayed high stiffness and strength in the dry state, with values of elastic modulus (*E*) and stress at break (*σ*_max_) approximately in the range 112–258 MPa and 4.6-7.7 MPa, respectively (Table 4), in rough accordance with previous studies on electrospun PHB-based mats [27, 42]. Noteworthy, both *E* and *σ*_max_ were found to be minimum for a PHB/Coll ratio of 70/30, whereas 100/0 and 50/50 membranes displayed similar values of *E* and *σ*_max_ (*p* = 0.13 for the modulus and *p* = 0.93 for the strength). Such a finding appears in partial disagreement with the crystallinity degrees calculated from the DSC analysis (Table 3), from which gradually decreasing values of *E* and *σ*_max_ would be expected for higher collagen content, and suggested that the mechanical stiffness and strength of the meshes were determined not only by PHB but also by collagen (in spite of its likely denaturation). If, on one side, the reduced stiffness of 70/30 fibrous mats, compared to 50/50 ones, may be partly attributable to their higher porosity (Table 2), it is also reasonable to assume that the collagen chains in the 50/50 samples are able to provide a significant amount of additional entanglements that act as physical crosslinking (and strengthening) nodes [15]. It is also worth noting that the measured *E* values suggest the potential application of the PHB/Coll mats as wound dressings or dermal substitutes, being their modulus roughly compatible with that of human skin (15–150 MPa) [43].

As regard to the elongation at break (*ε*_b_) of the mats, the average was in the range 13.5–29.5%, consistently with previous findings [10, 15, 34, 44]. In particular, *ε*_b_ was significantly reduced for samples containing collagen (PHB/Coll 70/30 and 50/50) compared to those with PHB only (*p* = 0.02 for 100/0 versus 50/50 and *p* = 0.001 for 100/0 versus 70/30, resp.), while no significant difference in *ε*_b_ was observed between 70/30 and 50/50 samples (*p* = 0.13). These findings seem to suggest that the additional molecular entanglements due to collagen lead to a less ductile behavior of the fibrous mats [39].

### 3.5. Degradation Rate

**Figure 7:**
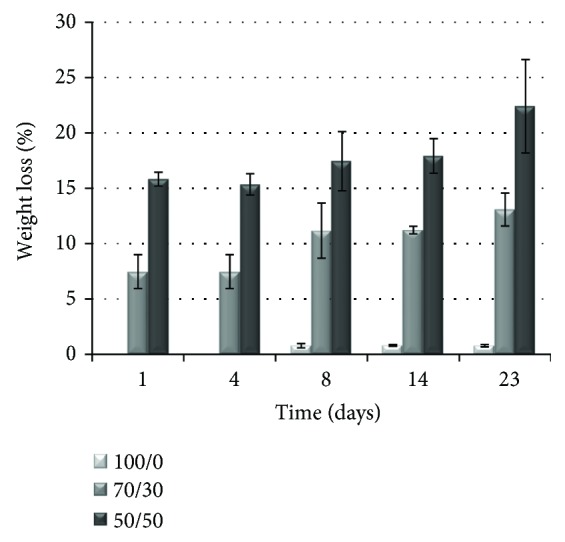
Hydrolytic degradation of the fibrous meshes, upon incubation in PBS at 37°C: weight loss percentage over time, as a function of the PHB/Coll ratio (mean ± SD, *n* = 3).

The assessment of the degradation kinetics is a fundamental step in the choice and/or development of biomaterials for tissue engineering. In nature, polyhydroxyalkanoates, such as PHB, are degraded by the action of nonspecific lipases and esterases [45]. *In vitro*, in the absence of enzymes, a slow hydrolysis process occurs [21, 27, 46]. As expected, all of the PHB-based mats preserved their structural integrity upon immersion in PBS at 37°C for 23 days. The recorded mass loss over time was indeed slight, although significantly dependent on the sample type, that is, on the PHB/Coll ratio (*p* < 0.0001). In particular, samples devoid of collagen were found to be the most resistant ones, with an average mass loss of 1% only after 23 days (Figure 7). This very slow degradation is likely ascribed to the slow surface erosion of hydrophobic PHB fibers due to hydrolysis, as well as to their high crystallinity, which further slows down the penetration of water in the amorphous regions. When increasing the collagen content in the polymer blend, the average mass loss of the samples increased, up to about 13% and 23% after 23 days, for PHB/Coll ratios of 70/30 and 50/50, respectively. Such a trend is in accordance with the hydrophilic character of the composite fibers, which is expected to induce a bulk erosion mechanism, and their lower crystallinity degrees, which facilitate water penetration. It is also worth noting that, based on the expected denaturation of collagen upon electrospinning, composite PHB/Coll mats are likely much more susceptible to hydrolysis than PHB ones, since the gelatin component (i.e., denatured collagen) is known to undergo a rapid hydrolytic degradation *in vitro* [47]. However, the overall high stability of the samples in water media confirmed that the blending of PHB and collagen is an effective way to avoid the rapid swelling and dissolution of fibrous collagen-based meshes in physiological fluids, without requiring additional crosslinking treatments.

The findings also demonstrated that, by changing the PHB/Coll ratio in the polymer blend, it is possible to adjust the degradation rate of the electrospun mats, in order to tailor specific tissue engineering applications, where the degradation rate of the scaffold should roughly match the rate of tissue regeneration. Nonetheless, it is important to highlight that the expected degradation profile of the PHB/Coll mats *in vivo* will be likely accelerated compared to the *in vitro* one. In addition to the enzymatic digestion of collagen, the *in vivo* degradation kinetics of PHB will be also affected by the catalytic action of nonspecific enzymes and cells [27]. Macrophages, usually located at the surface of PHB-based implants, are particularly able to phagocytize PHB and are also well known to secrete free radicals, acidic products, and enzymes that can further speed up the degradation of the polymer [27].

### 3.6. Cell Viability and Proliferation

**Figure 8:**
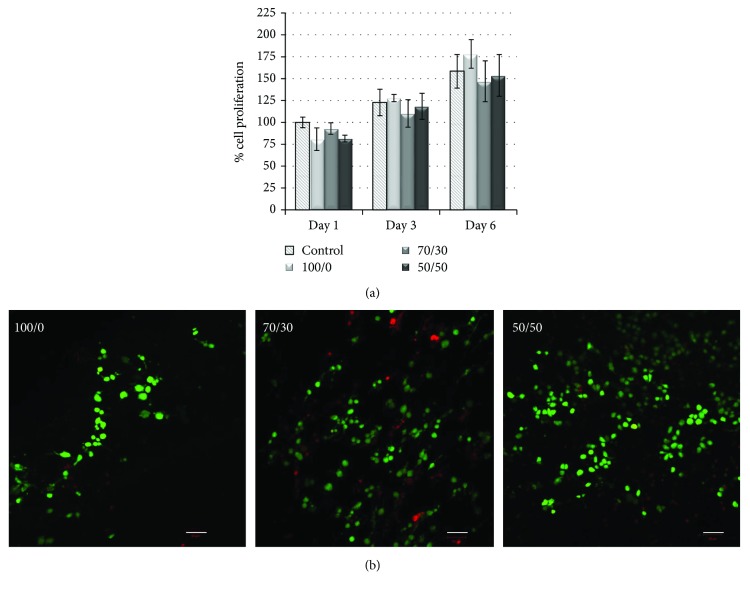
Viability and proliferation of 3T3 fibroblasts seeded on the PHB/Coll meshes: results of the MTT assay (mean ± SD, *n* = 3) at days 1, 3, and 6 postseeding (a) and confocal micrographs of FDA/PI staining at day 1 ((b) scale bar 50 *μ*m).

Murine fibroblasts were seeded on the PHB/Coll mats and kept in culture up to 6 days, in order to preliminarily assess any effects of the mats on cellular viability and proliferation. Cells grown on cell culture cover slides were taken as a reference. The results of the MTT assay (Figure 8a) showed that, at day 1, the viability of fibroblasts seeded on the mats was close to that of control cells, with no significant differences detected among the various experimental groups. The FDA/PI staining (Figure 8b) allowed estimating the percentage of viable (green) and nonviable (red) cells after 24 hours of culture and roughly confirmed the findings of the MTT assay at the same time point, indicating an average cellular viability of about 80% on all fibrous membranes.

As expected, significant cell proliferation was then observed at later time points (*p* < 0.0001 for culture time). In particular, at days 3 and 6, cell viability on reference and PHB/Coll samples increased of about 20–25% and 60–75%, respectively, with respect to day 1. At those time points, there were no significant differences in the percentage of cell proliferation between any of the fibrous samples and the control. This finding indicated a very good cell growth on the electrospun substrates, independently of their PHB/Coll ratio.

In the overall, the preliminary cellular experiment performed in this study suggested that all PHB/Coll membranes were able to support fibroblast attachment and proliferation. However, more accurate analyses should be performed to further investigate the expected contribution of collagen in the promotion of cell-material interactions, as well as to evaluate the actual capability of the mats to support the regeneration of selected tissues.

## 4. Conclusions

Blends of PHB and type I collagen, with different PHB/Coll weight ratios (100/0, 70/30, and 50/50), were successfully electrospun to obtain composite fibrous meshes with potential for tissue engineering applications. Experimental findings demonstrated that the addition of collagen to PHB, upon electrospinning, led to the formation of meshes with increased fiber diameter, improved wettability and thermal stability, decreased crystallinity, and enhanced sensitivity to hydrolytic degradation. Interestingly, the lowest mechanical stiffness and the highest porosity were detected for the 70/30 meshes. Despite the expected electrospinning-induced collagen denaturation and the reduced PHB crystallinity attained in the presence of collagen, mechanical data seemed to suggest a concurrent strengthening role of collagen in the composite samples, at least at high protein amounts, likely due to physical crosslinking of the collagen chains. The 50/50 meshes indeed showed values of elastic modulus and stress at break comparable to those of 100/0 ones. Further *in vitro* tests with murine fibroblasts highlighted that all of the fibrous substrates were highly cytocompatible and able to support cell growth, up to 6 days of culture. Although preliminary, this work put into evidence the potential of electrospun PHB/Coll mats as tunable substrates for tissue engineering and wound healing, with the PHB/Coll ratio controlling the morphological, mechanical, and degradation properties of the mats. Future studies will be directed to the synthesis of electrospun mats with preferential fiber orientation, which are particularly appealing as scaffolding materials for anisotropic tissues (e.g., nerve), as well as to more accurate investigations of cell-material interactions, in relation to the intended application.

## Figures and Tables

**Table 1:** Electrospinning parameters used for the synthesis of the different fibrous meshes.

PHB/Coll (*w/w*)	Voltage (kV)	Collection distance (cm)
100/0	12	12
70/30	14	22
50/50	16	24

**Table 2:** Thickness, porosity, and water contact angle (WCA) of the fibrous meshes as a function of the PHB/Coll ratio (mean ± SD; *n* = 3-4). For porosity, three values were calculated at different depths along the mesh thickness, corresponding to the surface (*P*_1_), middle (*P*_2_), and total porosity (*P*_3_).

PHB/Coll (*w/w*)	Thickness (*μ*m)	*P*_1_ (%)	*P*_2_ (%)	*P*_3_ (%)	WCA (°)
100/0	35 ± 1.7	84.3 ± 0.4	49.7 ± 1.6	17.2 ± 0.6	119.1 ± 10.2
70/30	50 ± 2.1^a^	83.7 ± 0.2	52.2 ± 0.4^a^	19.1 ± 0.7^a^	32.9 ± 10.6^∗^
50/50	39 ± 5.5	83.5 ± 0.4^a^	49.7 ± 0.9	17.8 ± 0.3	64.0 ± 12.8^a^

^a^Significant difference from PHB/Coll 100/0 (*p* < 0.05). ^∗^Nonnormal distribution of data.

**Table 3:** The results of DSC analysis: effect of electrospinning process and PHB/Coll ratio on PHB melting and degree of crystallinity (*χ*) (mean ± SD, *n* = 3-4).

	PHB/Coll (*w/w*)	*T*_m_ (°C)	Δ*H*_m_ (J/g)	*χ* (%)
PHB powder	100/0	175.4 ± 0.9	80.5 ± 1.1	55 ± 1
Fibrous mesh	100/0	176.7 ± 0.1^a^	94.6 ± 19.2	65 ± 13
	70/30	173.5 ± 0.9^a,b^	45.5 ± 1.7^a,b^	45 ± 2^b^
	50/50	171.8 ± 0.1^a,b^	27.3 ± 3.9^a,b^	37 ± 5^a,b^

^a^Significant difference from PHB powder (*p* < 0.05). ^b^Significant difference from PHB/Coll 100/0 (*p* < 0.05).

**Table 4:** Tensile properties of electrospun meshes: elastic modulus (*E*), stress at break (*σ*_max_), and elongation at break (*ε*_b_) as a function of the PHB/Coll ratio (mean ± SD, *n* = 5).

PHB/Coll (*w/w*)	*E* (MPa)	*σ*_max_ (MPa)	*ε*_b_ (%)
100/0	258.3 ± 7.9	7.7 ± 0.3	29.6 ± 7.5
70/30	116.0 ± 23.3^a^	4.7 ± 0.8^a^	13.5 ± 1.3^a^
50/50	225.4 ± 42.5	7.7 ± 1.4	19.6 ± 2.4^a^

^a^Significant difference from PHB/Coll 100/0 (*p* < 0.05).
